# Ubiquitin-Conjugating Enzyme UBE2C Is Highly Expressed in Breast Microcalcification Lesions

**DOI:** 10.1371/journal.pone.0093934

**Published:** 2014-04-03

**Authors:** Chen-Pin Chou, Nan-Chieh Huang, Shu-Jhen Jhuang, Huay-Ben Pan, Nan-Jing Peng, Jiin-Tsuey Cheng, Chian-Feng Chen, Jih-Jung Chen, Tsung-Hsien Chang

**Affiliations:** 1 Department of Radiology, Kaohsiung Veterans General Hospital, Kaohsiung, Taiwan; 2 School of Medicine, National Yang-Ming University, Taipei, Taiwan; 3 Department of Family Medicine, Zuoying Branch of Kaohsiung Armed Forces General Hospital, Kaohsiung, Taiwan; 4 Department of Pathology and Laboratory Medicine, Kaohsiung Veterans General Hospital, Kaohsiung, Taiwan; 5 Department of Medical Education and Research, Kaohsiung Veterans General Hospital, Kaohsiung, Taiwan; 6 Department of Biological Sciences, National Sun Yat-Sen University, Kaohsiung, Taiwan; 7 Department of Medical Imaging and Radiological Sciences, I-Shou University, Kaohsiung, Taiwan; 8 Section of Nuclear Medicine, Kaohsiung Veterans General Hospital, Kaohsiung, Taiwan; 9 VYM Genome Research Center, National Yang-Ming University, Taipei, Taiwan; 10 Graduate Institute of Pharmaceutical Technology, Tajen University, Pingtung, Taiwan; University of North Carolina School of Medicine, United States of America

## Abstract

Ubiquitin-conjugating enzyme 2C (UBE2C) contributes to ubiquitin-mediated proteasome degradation of cell cycle progression in breast cancer. Microcalcification (MC) is the most common mammographic feature of early breast cancer. In this study, we evaluated whether UBE2C could be a tumor marker of early breast cancer with MC found on screening mammography. UBE2C protein and mRNA expression were measured in breast core biopsy pairs of MC and adjacent non-MC breast tissue from each subject. Immunohistochemistry revealed UBE2C positivity in 69.4% of MC samples and 77.6% negativity in non-MC samples (p<0.0001). On RT-qPCR, 56.1% of malignant MC lesion samples showed high mRNA level of UBE2C and 80% of benign MC lesion samples showed a low level of UBE2C (p = 0.1766). We investigated the carcinogenic role of UBE2C in MCF-7 breast cancer cells with UBE2C knockdown; UBE2C knockdown downregulated cell proliferation and activated the cellular apoptosis pathway to inhibit cell colony formation. Furthermore, UBE2C expression was associated with that of carcinogenic genes human epidermal growth factor receptor type 2 (HER2), cellular c-Ki-ras2 proto-oncogene (KRAS), vascular endothelial growth factor (VEGF), CXC chemokine receptor 4 (CXCR4), C-C motif chemokine 5 (CCL5), neural precursor cell expressed, developmentally downregulated 9 (NEDD9) and Ras homolog family member C (RhoC). UBE2C may be a marker for diagnosis of nonpalpable breast lesions but not benign or malignant tumors in mammography core biopsies. Suppression of UBE2C may be a potential therapy target in breast cancer.

## Introduction

Breast cancer represents the highest cancer incidence rate and the fourth highest mortality rate for women in Taiwan [1], [2]. Early diagnosis and proper treatment are critical in patient survival [3], [4], [5]. Several tests performed to stage breast cancer include biopsy and imaging tests such as chest x-ray, mammography, bone scan, CT and MRI [6]. Mammography is the most important imaging tool for the detection and diagnosis of breast cancer, particularly for non-invasive ductal carcinoma in situ (DCIS) breast cancer [7]. Screening mammography can detect early, nonpalpable breast cancer, because as many as 2% of all screened women will undergo biopsy, thus yielding a positive biopsy rate of about 25% and improved long-term survival and cure rate [8]. Since 2004, Taiwan began a phased implementation of screening mammography every 2 years for women aged 50 to 69. More than half of the breast cancer cases found were at highly curable stage 0 and 1 [2].

Breast microcalcifications (MC), detected by mammography, result from inflammation of the breast, intraductal papilloma, fibroadenoma, cystic fibrosis, fat necrosis and MC with breast cancer, because calcification itself can be a normal or abnormal cell-death metabolic physiological phenomenon or the result of cancer [9]. Mammography-sterotactic vacuum-assisted core needle biopsy allows for minimally invasive sampling of most MC breast lesions and generates tissue cores for histologic evaluation. Such biopsy samples are also valuable for investigating tumor markers of early breast cancer.

Tumor biomarker tests have additional features for breast cancer diagnosis and treatment. The expression of immunohistochemical markers of human epidermal growth factor receptor 2 (HER2/neu), estrogen receptor (ER) and progesterone receptor (PR) have been widely accepted for routine use in breast cancer, serving as predictive factors of endocrine and trastuzumab therapy, respectively [10], [11]. However, the weak positivity of HER2, ER and PR or triple-negative breast cancer (HER2-/ER-/and PR-) requires additional examination with costly and higher technical assays, such as fluorescence *in situ* hybridization assay [11], [12], [13]. Therefore, developing new biomarkers for diagnosis and examining the genomic diversity of breast cancer is needed.

Ubiquitination is a critical cellular mechanism for targeting abnormal or short-lived proteins for degradation. The modification of proteins with ubiquitin involves at least 3 classes of enzymes: ubiquitin-activating enzymes, ubiquitin-conjugating enzymes (E2s), and ubiquitin-protein ligases. Ubiquitin-conjugating enzyme 2C (*UBE2C*) encodes a member of the E2 ubiquitin-conjugating enzyme family; the gene features multiple transcript variants encoding different isoforms. UBE2C is required for destroying mitotic cyclins and regulating anaphase-promoting complex and for cell cycle progression [14], [15]. Overexpression of UBE2C causes chromosome missegregation and alters the cell cycle profile, which facilitates cell proliferation [16], [17]. Importantly, UBE2C transgenic mice show a broad spectrum of spontaneous tumors that demonstrate UBE2C as a prominent protooncogene [17]. UBE2C expression is associated with aggressive thyroid, ovarian and breast carcinomas; colon cancer; and lymphomas [18], [19], [20], [21], [22], [23]. It may be a tumor marker candidate.

To evaluate the practical application of UBE2C in early breast cancer diagnosis, we measured UBE2C mRNA and protein expression in vacuum-assisted breast core biopsies and analyzed the relation between UBE2C expression and clinical features. We also investigated the carcinogenic role of UBE2C in the breast cancer cell cycle by its overexpression or knockdown ectopic expression. In addition, we examined the expression of UBE2C-related tumor-initiation and metastasis genes. We provide a theoretical basis for UBE2C as a potential biomarker candidate or theraputic target for early breast cancer.

## Material and Methods

### Ethic statement

The study was approved by the Institutional Review Board of Kaohsiung Veterans General Hospital (Protocol number: VGHKS11-CT4-02) and conformed to the current ethical principles of the Declaration of Helsinki. Written informed consents were obtained from all subjects.

### Human tissue samples and cell lines

Women with Breast Imaging Reporting and Data System (BI-RADS) category 4 or 5 due to suspicious malignant MC on screening or diagnostic mammography were recommended for stereotactic breast biopsy. Biopsies of breast MC and adjacent normal tissue (non-MC) were obtained by the stereotactic 10-gauge vacuum-assisted breast biopsy system (Vacora, Bard Medical Systems, Tempe, AZ). In general, 15 to 20 specimens per lesion were obtained. Women were included if mammography between January 2010 and December 2011 showed that MC specimens were insufficient for clinical pathology diagnosis.

Breast cancer cell lines, MCF-7 (BCRC#60436) and MDA-MB-231 (BCRC#60549) from the Bioresource Collection and Research Center (BRCR, Hsinchu, Taiwan) were cultured in DMEM supplemented with 10% fetal bovine serum (Invitrogen) in a humidified atmosphere of 95% air and 5% CO_2_ at 37°C.

### Immunohistochemistry

Formalin-fixed, paraffin-embedded breast core biopsies were cut into 5-μm sections and mounted on slides. Following deparaffinisation in xylene, slides were derehydrated in a alcohol graded series and placed in running water. The Novolink Polymer Detection System (Leica) was used for immunohistochemistry. The antigen was retrieved with heating in 10 mM citrate buffer (pH 6.0), then slides were incubated with Peroxidase Block to neutrilize endogenouse peroxidase activity, then with Protein Block before reaction with anti-UBE2C antibody (1∶100, H00011065-M01, Abnova). Then, slides were reacted with Novolink polymer followed by DAB chromogen solution to develop peroxidase activity for visualizing the antibody–drochloride complex. Slides were counterstained with haematoxylin. UBE2C histology score was defined as the proportion of immunostained cells to total number of cells in the evaluated area. A trace amount (+/−) indicated <10% nuclear staining; 1+, 10% to 30% cells with faint or barely perceptible staining; 2+, 30% to 50% cells with strong staining; 3+, >50% cells with strong staining; and 4+, almost 100% cells with strong staining.

### RNA extraction and quantitative real-time PCR

Total RNA was extracted from biopsies or cultured cells by the Trizol reagent method (Invitrogen). In total, 1 μg total RNA was reverse-transcribed with oligo dT primer and the Superscript III reverse transcription kit (Invitrogen). Quantitative RT-PCR involved 4 ng cDNA with 10 μl SYBR Green PCR Master Mix (Applied Biosystems) and 3 μM primers in the ABI StepONE Real-Time PCR System (Applied Biosystems). The primers for UBE2C, cellular c-Ki-ras2 proto-oncogene (KRAS), HER2, vascular endothelial growth factor (VEGF), CXC chemokine receptor 4 (CXCR4), C-C motif chemokine 5 (CCL5), neural precursor cell expressed, developmentally downregulated 9 (NEDD9) and Ras homolog family member C (RhoC) were designed by use of Primer Express v3.1 (Applied Biosystems; Table S1). The relative gene mRNA expression was normalized to that of HPRT as a loading control.

### Western blot analysis

For protein extraction, 1×10^6^ cells were lysed in 200 μl RIPA buffer (50 mM Tris-HCl [pH 7.5], 150 mM NaCl, 1 mM EDTA, 1% Nonidet P-40, 0.5% DOC, 0.1% SDS) containing Complete Protease Inhibitor Cocktail (Roche). Cell lysates were centrifuged at 14,000 × g for 30 min at 4°C, then supernatant was harvested. Proteins were quantified by use of the Bio-Rad DC Protein Assay kit, separated by 10% SDS-PAGE and transferred to PVDF membrane (Millipore). Membranes were blotted with antibodies for UBE2C (1∶1000, H00011065-M01, Abnova), Bcl-2 (1∶1000, 1017-1, Epitomics), and poly (ADP-ribose) polymerase (PARP; 46D11), Bcl-XL (2762), caspase-3 (8G10), caspase-8 (D35G2) and caspase-9 (all 1∶1000, C9, all Cell Signaling). Incubation with anti-β-actin (1∶5000, MAB1501, Millipore) was a loading control.

### Overexpression and knockdown of UBE2C

UBE2C cDNA was cloned from MCF-7 cells by use of PCR primers with XhoI and EcoRI restriction enzyme sites. The primer sequence for UBE2C was forward, ATCTCGAGTGTTCTCCGAGTTCCTGTC, and reverse, GTGAATTCTCAGGGCTCCTGGCTG. The cloned UBE2C cDNA was inserted into the expression vector pMSCV(-puro) (Clontech), then sequenced. An amount of 4 μg pMSCV-UBE2C plasmid was transfected into MCF-7 cells by PolyJet transfection (SignaGen Laboratories). At 48 h post-transfection, overexpressed UBE2C was detected by immunoblotting. To knock down UBE2C expression in MCF-7 cells, UBE2C short hairpin RNA (shRNA) and control shRNA oligonucleotides were synthesized and inserted into the pSUPER-retro-vector (Oilogoengin) to generate UBE2C shRNA. The primer sequences were for UBE2C shRNA, forward, GATCCCCCCTGCAAGAAACCTACTCATTCAAGAGATGAGTAGGTTTCTTGCAGGTTTTTA, and reverse, AGCTTAAAAACCTGCAAGAAACCTACTCATCTCTTGAATGAGTAGGTTTCTTGCAGGGGG; and control shRNA, forward, GATCCCCCTAACACTAGCTCAAGACCTTCAAGAGAGGTCTTGAGCTAGTGTTAGTTTTTA and reverse, AGCTTAAAAACTAACACTAGCTCAAGACCTCTCTTGAAGGTCTTGAGCTAGTGTTAGGGG. An amount of 4 μg pSUPER-UBE2C and control shRNA plasmids was transfected into MCF-7 cells for 48 h for expression analysis.

### Cell viability and proliferation assay

MTT and WST-1 assay were used to monitor the viability and proliferation, respectively, of MCF-7 cells. For MTT assay, MCF-7 cells were trypsinized and resuspended in culture medium, then plated at 5×10^3^ cells per well in 96-well plates and incubated overnight. MCF-7 cell viability was determined by 3-(4,5-dimethylthiazol-2-yl) 2,5-diphenyltetrazolium bromide MTT reagent at 1 mg/ml for 30 min in 96-well plates, then the cell supernatant was replaced with DMSO to resolve formazan dye; viability was quantified by scanning multi-well spectrophotometry (Anthos). The net absorbance (OD550 to OD620 nm) was measured. The dye exclusion test for cell viability involved Trypan blue staining (Gibco). For WST-1 assay, cells grown in 96-well plates were incubated with 10 μl WST-1 reagent (Roche) for 2 h. The absorbance at 450 nm was monitored and the reference wavelength was set at 620 nm.

### Wound healing assay

MCF-7 cells were cultured in confluent monolayers in 12- or 6-well plates. The monolayers were scratched in a line across the well with use of a 200-μl standard pipette tip. The wounded monolayers were then washed twice with serum-free media to remove cell debris and incubated. The cell-free wound area was photographed at the indicated times with use of a digital camera connected to an inverted microscope (Nikon TE200). Images were analyzed by use of Image J. Wound healing was calculated as the proportion of remaining cell-free area compared with the initial wound area.

### Colony-forming assay

MCF-7 cells were cultured at 2,000 cells/well in 6-well plates. The cells were allowed to grow for 14 days, with medium changed every 3 days. At the end, colonies were fixed with paraformadehyde (4% w/v), stained with crystal violet (0.5% w/v) and counted.

### Statistical analysis

Statistic analyses involved use of GraphPad Prism 5 (GraphPad Software, Inc., La Jolla, CA, USA). Data were analyzed by Wilcoxon matched-pairs *t* test, Mann-Whitney U test, receiver-operating characteristic curve analysis, chi-square test or Fisher's extract test. Correlation analysis involved Pearson correlation coefficient. Two-tailed *P*≤0.05 was considered statistically significant.

## Results

### Immunohistochemical analysis of UBE2C expression in breast MC tissues

Mammographically determined MC lesions from core biopsies of suspected breast cancer were confirmed by pathology diagnosis. We included samples from 55 women, from 27 to 70 years old; pathology diagnoses included 21 benign (38%) and 34 malignant (62%) cases. Among the 34 malignant cases, 19 were invasive ductal carcinoma (IDC) and 14 DCIS (Table 1). According to tumor-node-metastasis staging, most of the malignant cases were stage 0 (n = 13, 38%) and I–II (n = 19, 56%).

**Table 1:** Data for 55 women.

Clinical parameter	No. (%)
**History**	
Personal history of breast cancer	7 (13)
Family history of breast cancer	2 (4)
**Age**	
≧50	38 (69)
<50	17 (31)
**Mammography BI-RADS category**	
4A	20 (36)
4B	12 (22)
4C	11 (20)
5	12 (22)
**Pathology type**	
Malignant	34 (62)
Benign	21 (38)
**TNM cancer stage (34 malignancy)**	
0	13 (38)
I–II	19 (56)
III–IV	2 (6)
**Histology**	
**Invasive cancer**	
Invasive ductal carcinoma	19 (35)
**Carcinoma in situ**	
Ductal carcinoma in situ	14 (25)
Lobular carcinoma in situ	1 (2)
**Benign**	
Atypical ductal hyperplasia	1 (2)
Flat epithelial atypia	3 (5)
Fibrocystic disease	17(31)

TNM, tumor-node-metastasis.

**Figure 1:**
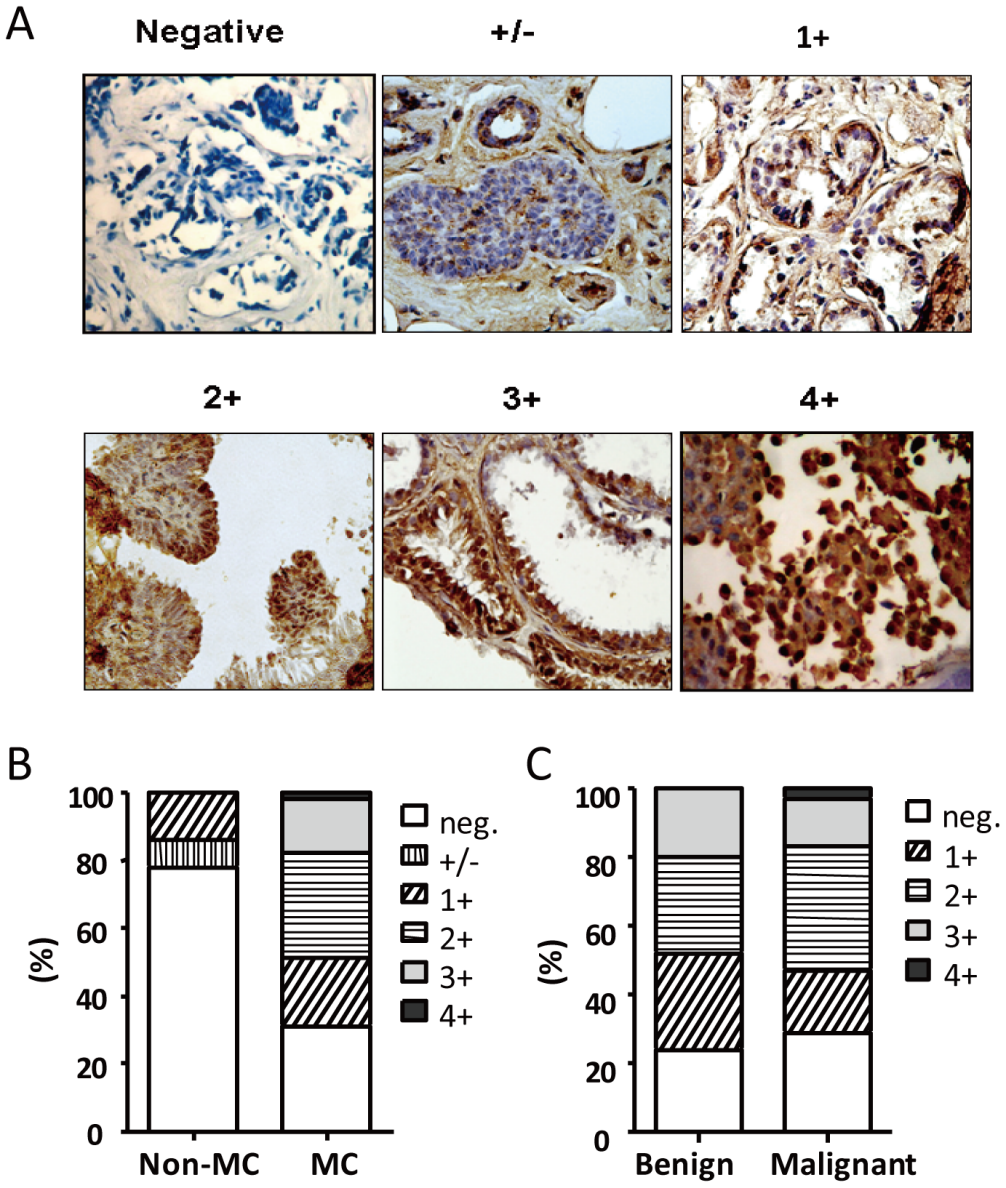
Immunohistochemistry of UBE2C expression in core breast biopsies. (**A**) Immunohistochemistry staining for UBE2C protein expression: staining score from 0 (negative) to 4+ (positive). (**B**) UBE2C scores in breast non-microcalcification (non-MC) and microcalcification (MC) tissues. (**C**) UBE2C scores for benign and malignant samples by pathological diagnosis.

Six specimens were not included in our analysis because of a limited specimen for good-quality staining. Finally, 49 pairs of breast non-MC and MC biopsies underwent immunhistochemistry for UBE2C expression (Figure 1A). UBE2C expression was high in MC samples (score 1+, 20%; 2+, 31%; 3+, 16%; and 4+, 2%) but not in non-MC samples (+/−, 8%, 1+, 14%, *P*<0.0001; Figure 1B). However, the scores for UBE2C were ambiguous between benign and malignant MC lesions (Figure 1C).

In total, 69.4% of MC specimens were UBE2C-positive and only 22.4% of non-MC specimens were UBE2C-positive; with no difference in UBE2C staining by age, BI-RADS, or pathology type or stage (Table 2). The immunohistochemistry findings suggested that UBE2C expression might be associated with breast MC lesions, but we need a larger cohort to clearly define the implications of UBE2C expression in benign and malignant tumors.

**Table 2:** Immunohistochemistry analysis of ubiquitin-conjugating enzyme 2C (UBE2C) in breast lesions by clinical variables.

		UBE2C		
Variable	No. of samples	Negative, no. (%)	Positive, no. (%)	*P*-value^b^
**Lesion type** a				
Non-MC	49	38 (77.6)	11 (22.4)	
MC	49	15 (30.6)	34 (69.4)	**<00001**
**Age**				
≧50	33	10 (30)	23 (70)	
<50	16	5 (31.25)	11 (68.75)	1.000
**BI-RADS**				
4A	20	5 (25)	15 (75)	
4B	11	4 (36.4)	7 (63.6)	
4C	9	1 (11.1)	8 (88.9)	
5	9	5 (55.6)	4(44.4)	0.1939
**Pathology type**				
Benign	21	7 (33.3)	14 (66.7)	
Malignant	28	8 (28.6)	20 (71.4)	0.7621
**TNM cancer stage**				
DCIS/LCIS stage 0	12	1 (8.3)	11 (91.7)	
IDC stage I–III	16	7 (43.8)	9 (56.2)	0.0826

a Mammography diagnosis of non-microcalcification (Non-MC) and microcalcification (MC) tissue. The MC results were further analyzed by age, Breast Imaging Reporting and Data System (BI-RADS) category, pathology type and cancer stage.

b determined by Fisher's exact test, except BI-RAD, which was analyzed by chi-square test.

DCIS/LCIS, ductal carcinoma in situ*/lobular carcinoma* in situ; IDC, invasive ductal carcinoma.

### Relationship between clinicopathologocal characteristics and UBE2C mRNA expression in breast MC lesions

**Figure 2:**
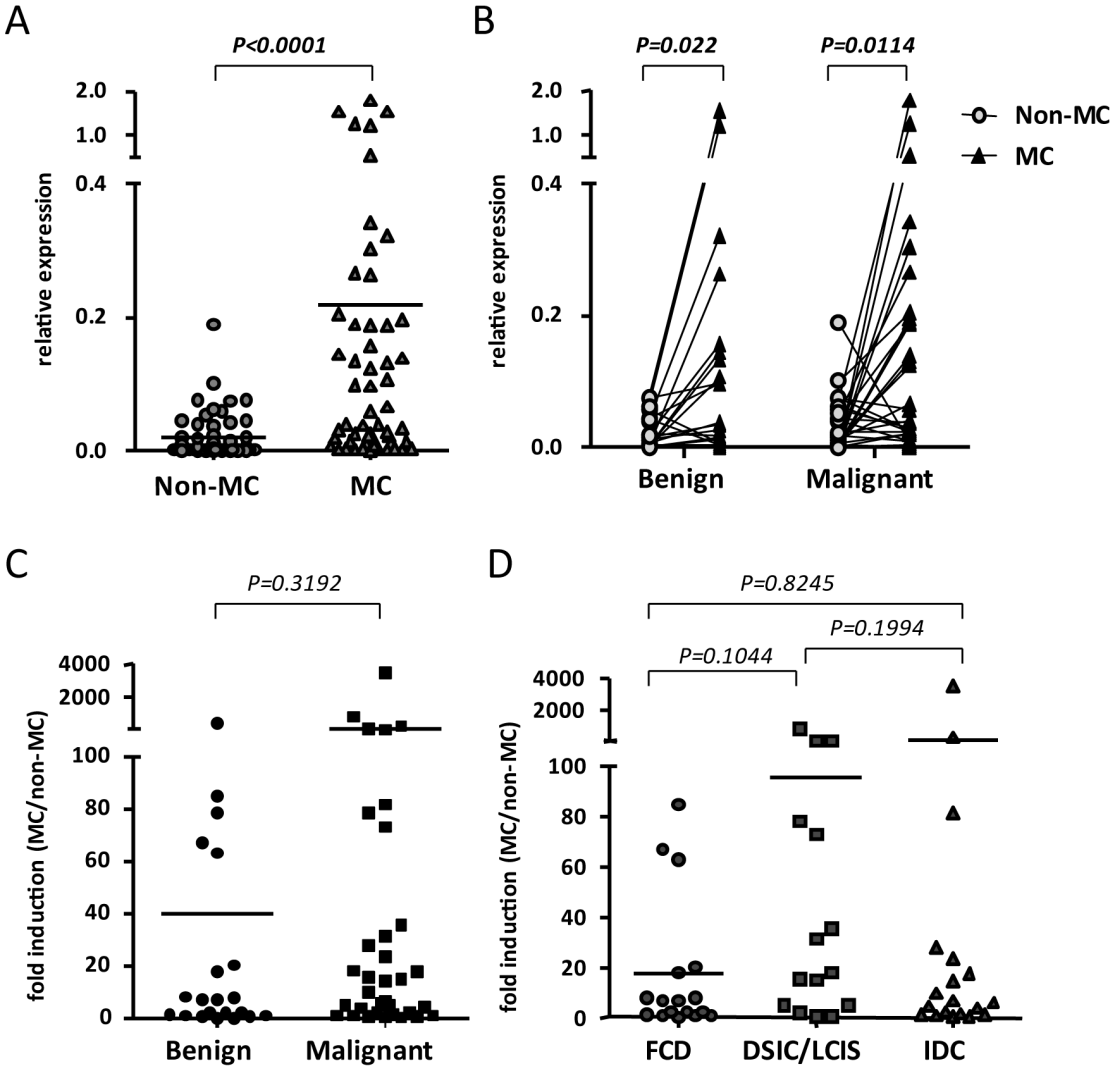
UBE2C mRNA expression and clinico-pathological data. (**A**) UBE2C relative expression in non-MC and MC tissue with normalization to the internal control HPRT. (**B**) The relative expression of UBE2C mRNA in pairs of non-MC and MC lesions in benign and malignant samples. (**C**) UBE2C fold change in mRNA expression in MC/non-MC lesions in pairs of benign and malignant samples. (**D**) UBE2C fold change in mRNA expression in MC/non-MC lesions in fibrocystic disease (FCD), ductal carcinoma in situ (DSIC)/lobular carcinoma in situ (LCIS) and invasive ductal carcinoma (IDC) samples. Data are mean±SEM.

We measured UBE2C mRNA expression in 55 pairs of biopsies. UBE2C expression was greater in MC than non-MC lesions (*p*<0.0001; Figure 2A); benign and malignant samples showed a similar pattern of high UBE2C expression in MC lesions (Figure 2B). UBE2C relative expression (MC/non-MC) was greater but not significantly in malignant than benign samples (Figure 2C). Pathology diagnosis showed similar results, with lower UBE2C expression with benign fibrocystic disease than DCIS/lobular carcinoma *in situ* and IDC malignant lesions (Figure 2D).

With a two-fold optimal cut-off (25th percentile) of UBE2C expression, 76.5% and 66.7% of malignant and benign samples, respectively, showed a two-fold increase in UBE2C expression, although not statistically significant (Table 3). With a nine-fold optimal cut-off of UBE2C expression from receiver-operating characteristic curve analysis (52.9% sensitivity and 66.4% specificity), 56.1% and 20% of malignant and benign samples, respectively, showed a nine-fold increase in UBE2C mRNA expression. The result was similar by age, cancer stage and tumor size, with no difference between groups (Table 4). Therefore, even though MC lesions expressed high UBE2C mRNA level, UBE2C could not be a diagnostic biomarker of malignant breast cancer in mammography core biopsies, which agrees with immunohistochemistry data (Table 2).

**Table 3:** Association of two-fold change in UBE2C mRNA level (MC/non-MC lesions) and pathologic features.

		UBE2C fold induction (MC/non-MC)		
Pathology type	No. of samples	≥2-fold, no. (%)	<2-fold, no. (%)	*P*-value^a^
Benign	21	14 (66.7)	7 (33.3)	
Malignant	34	26 (76.5)	8 (23.5)	0.5366
Total	55	40 (72.7)	15 (27.3)	

a Two-sided *P*-value by Fisher's exact test.

**Table 4:** Association of nine-fold change in UBE2C mRNA level (MC/non-MC lesions) and pathologic and clinical features.

		UBE2C fold induction (MC/non-MC)^a^		
Parameter	No. of samples	≥9-fold, no. (%)	<9-fold, no. (%)	*P*-value^b^
**Pathology type**				
Benign	21	7 (20)	14 (80)	
Malignant	34	18 (56.1)	16 (43.9)	0.1766
**Age** c				
≧50	22	12 (54.5)	10 (45.5)	
<50	12	6 (50)	6 (50)	1.0000
**TNM stage**				
0	14	10 (71.4)	4 (28.6)	
I–III	20	8 (40)	12(60)	0.0921
**Tumor size** d				
T1	14	6 (42.9)	8 (57.1)	
T2∼T3	7	3 (42.9)	4 (57.1)	1.0000

a Receiver-operating characteristic curve determined nine-fold induction of UBE2C mRNA level as the cut-off showing 52.9% sensitivity and 66.7% specificity.

b Two-sided *P*-value by Fisher exact test.

c Patients with malignant breast cancer.

d Tumor size typing of IDC.

We determined the expression of the common breast tumor markers HER2, ER and PR in MC biopsies with IDC by immunohistochemistry and then analyzed the fold induction of UBE2C. Samples with high expression of HER2, ER and PR also showed high expression of UBE2C mRNA, although these data need a larger cohort for statistical significance analysis (Figure S1A–C).

### UBE2C is required for breast cancer cell growth

**Figure 3:**
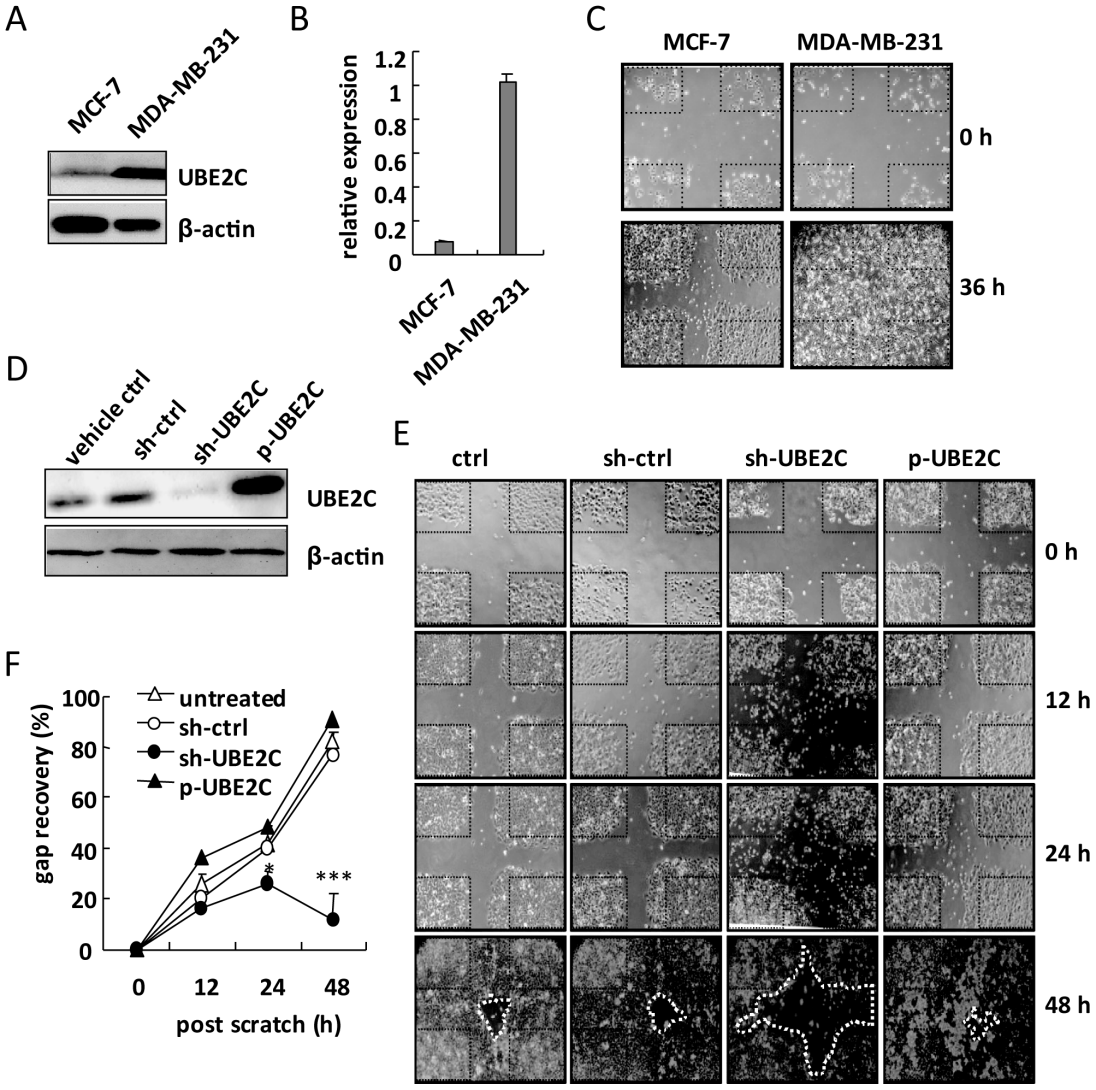
Effect of UBE2C expression on breast cancer cell proliferation and migration. (**A**) Western blot analysis of UBE2C expression in MCF-7 and MDA MB-231 cells. β-actin was an internal control. (**B**) RT-qPCR analysis of UBE2C mRNA expression in MCF-7 and MDA MB-231 cells. The internal control was HPRT. Data are mean ± SD from 3 tests. (**C**) Wound-healing test in MCF-7 and MDA-MB-231 cells grown in 12-well plates until 80% confluence, then cells were scratched in a line; images were obtained under an inverted optical microscopy. (**D**) MCF-7 cells were transfected with the UBE2C overexpression plasmid, p-UBE2C, or UBE2C knockdown or control plasmids sh-UBE2C or sh-ctrl for 48 h. Western blot analysis of protein level of UBE2C and β-actin. (**E**) Wound-healing assay of transfected MCF-7 cells at 0, 12, 24 and 48 h. The circled area with a dotted line was analyzed for gap recovery rate. (**F**) Gap recovery rates obtained from 3 independent wound-healing assays. Data are mean ± SD; *P<0.05, ***P<0.0001 vs. sh-ctrl.

Because we found that UBE2C induction might play a role in breast lesions, although the link with malignant tumor progression was ambiguous, we investigated the carcinogenic role of UBE2C in the human breast cancer cell lines MCF-7 and MDA-MB-231. Compared with MDA-MB-231 cells, MCF-7 cells expressed lower levels of UBE2C protein and mRNA (Figure 3A and B); this result was supported in NCI-60 human cancer cells database (data not shown). We investigated the difference in cell proliferation and migration between MCF-7 and MDA-MB-231 cells by wound-healing assay. As expected, MDA-MB-231 cells showed greater cell proliferation and wound recovery than did MCF-7 cells (Figure 3C).

To understand the effect of UBE2C in breast cancer cell growth and migration, we overexpressed or knocked down UBE2C in MCF-7 cells. Western blot analysis confirmed the results of overexpression or knockdown (Figure 3D). As compared with control cells, UBE2C knocked-down cells showed defective wound healing; and UBE2C overexpression only modestly improved wound healing (Figure 3E and F). Therefore, UBE2C may be essential for MCF-7 cell growth, and the level of endogenous UBE2C was sufficient for cell growth and migration.

### UBE2C knockdown triggers apoptosis

**Figure 4:**
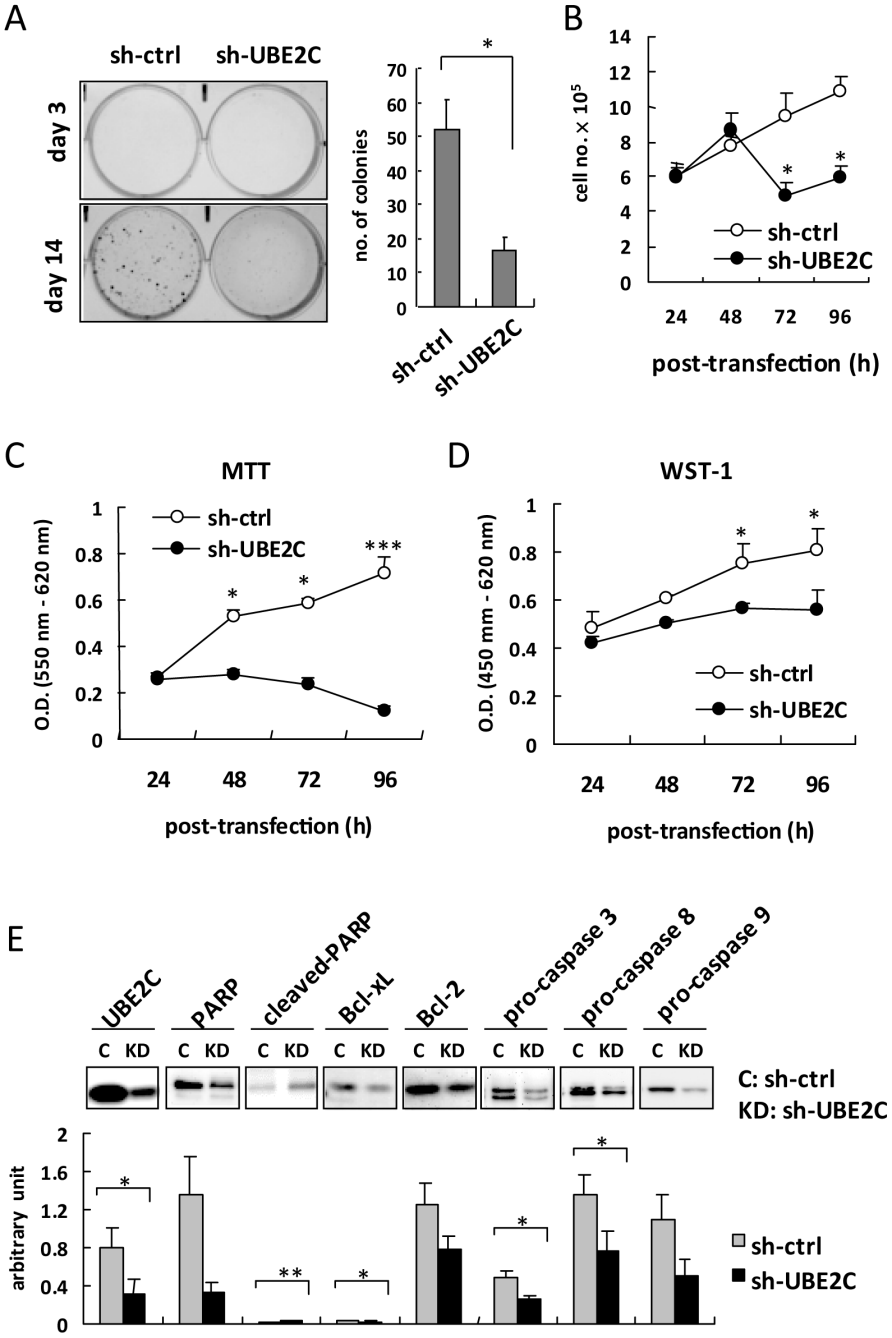
Cell growth reduction and apoptosis pathway activation in MCF-7 cell with UBE2C knockdown. (**A**) Colony-forming assay in MCF-7 cells with UBE2C shRNA knockdown or control shRNA at days 3 and 14 (left panel). Data are mean ± SD from 3 independent tests. *P<0.05 (right panel). (**B**) Trypan blue exclusion test of survival of MCF-7 cells transfected with UBE2C knockdown or control shRNA. *P<0.05 vs. sh-ctrl. (**C**) MTT viability assay and (**D**) WST-1 proliferation assay in MCF-7 cells with UBE2C shRNA knockdown or control shRNA at the indicated times. Data are mean ± SD from triplicate tests. *P<0.05 ***P<0.001. (**E**) Western blot analysis of apoptosis or anti-apoptosis protein levels in MCF-7 cells transfected with UBE2C knockdown (KD: sh-UBE2C) or control shRNA (C, sh-ctrl) for 72 h. Quantitative arbitrary unit of blots was normalized to β-actin (lower panel). Data are mean ± SD from 3 independent tests. *P<0.05 **P<0.005.

We further investigated the carcinogenic activity of UBE2C by colony-forming assay in MCF-7 cells with UBE2C knockdown. Cells with UBE2C knockdown showed suppressed colony formation after 14 days of growth (Figure 4A). The effect of UBE2C in MCF-7 cell viability was determined by quantification of Trypan blue staining (Figure 4B), MTT assay (Figure 4C) and WST-1 assay (Figure 4D). MCF-7 cells with UBE2C knockdown showed reduced cell viability. These data indicate the activation of cellular apoptosis with UBE2C knockdown. Western blot analysis of apoptotic protein expression revealed decreased protein level of full-length PARP, Bcl-xL, Bcl-2, and pro-caspase-3, -8, and -9 and increased level of cleaved-PARP in UBE2C knockdown but not control cells (Figure 4E, upper panels); the normalized immunoblot arbitrary unit with β-actin gave consistent results (Figure 4E, lower panel). Thus, UBE2C inhibition caused transduction of apoptosis molecules. UBE2C may be essential for MCF-7 cell colony-forming activity and preventing apoptosis. UBE2C may be a therapeutic target for breast cancer.

### Expression of UBE2C-associated carcinogenic genes

**Figure 5:**
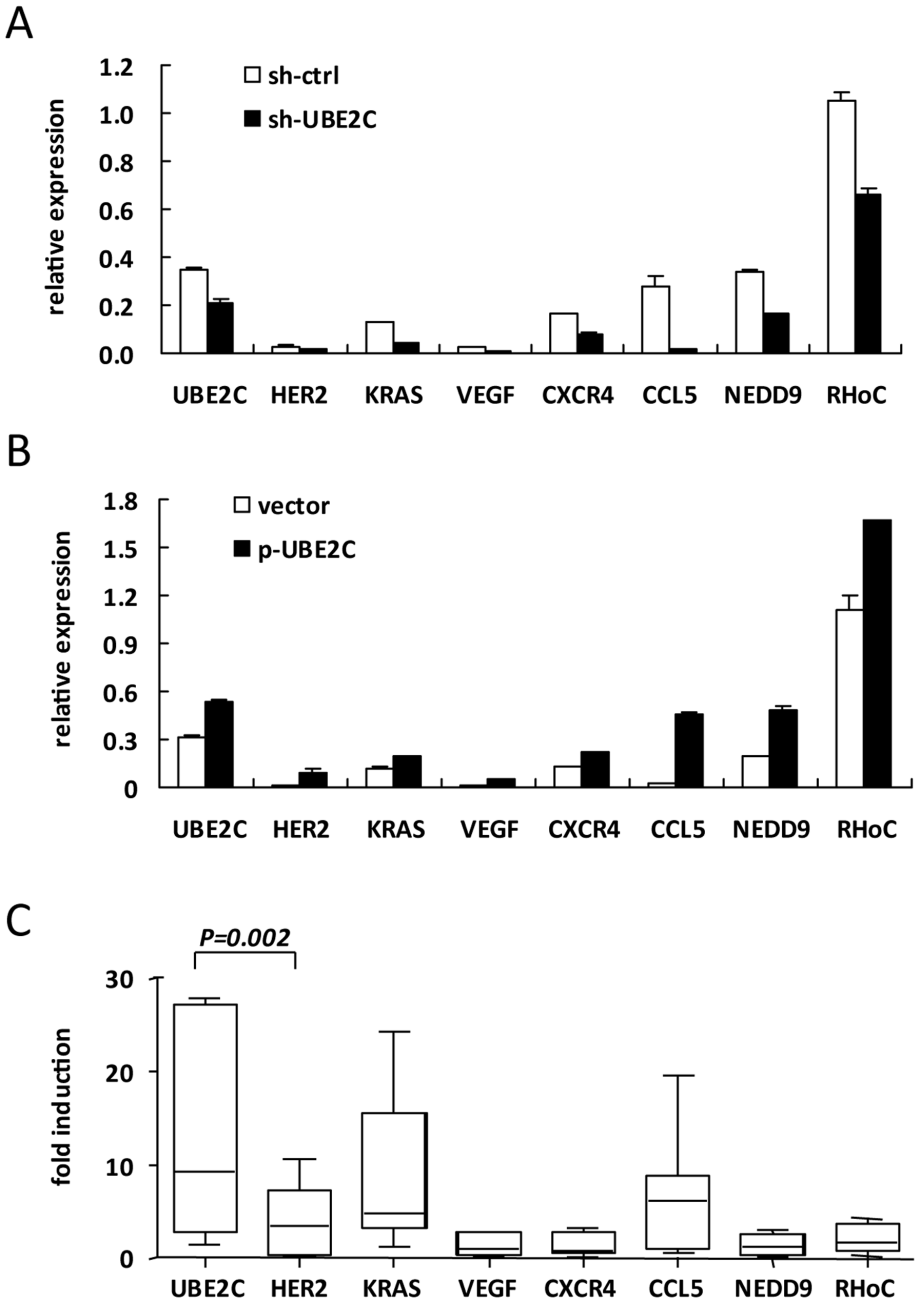
Expression of UBE2C associated with that of oncogenic genes. RT-qPCR analysis of mRNA level of UBE2C and selected tumor-initiation or migration genes in MCF-7 cells with UBE2C knockdown, sh-UBE2C (**A**), or UBE2C overexpression, p-UBE2C (**B**) The relative mRNA expression was normalized to the internal control, HPRT. (**C**) RT-qPCR analysis of mRNA expression in 9 pairs of MC-malignant and non-MC core biopsies. UBE2C and HER-2 correlation: r^2^ = 0.879, *P* = 0.002.

Human neoplasias derive from genetic alteration inside cells. This alteration results in marked changes in levels of proteins involved in cell growth control, signal transduction and the cellular regulatory system in a specific and characteristic manner [24]. We wondered whether UBE2C expression was associated with that of other oncogenic genes in breast cancer cells. According to a previous review [25], we measured the mRNA expression of 5 tumor initiation genes and 20 metastasis initiation, progression and virulence genes in MCF-7 cells with UBE2C knockdown. The levels of the tumor initiators HER2 and KRAS and metastasis genes VEGF, CXCL-4, CCL5, NEDD9 and RHoC were reduced in MCF-7 cells with UBE2C knockdown (Figure 5A) and increased with UBE2C overexpression (Figure 5B). Therefore, expression of the 7 carcinogenic genes changed with UBE2C expression in MCF-7 cells. To understand whether expression of these UBE2C-related genes was unregulated in malignant MC specimens, we randomly selected 9 MC breast biopsy pairs and found increased induction of HER2, KRAS and CCL-5 expression; HER-2 expression was highly correlated with UBE2C (r^2^ = 0.879, *P* = 0.002; Figure 5C). Therefore, the expression of UBE2C was associated with that of selected carcinogenic genes in breast cancer cells and core biopsies and might be involved in breast cancer tumor formation or migration.

## Discussion

Mammography screening is effective in detection of early breast tumors in Taiwan women [26]. After mammography detection, core biopsy specimens of breast lesions can be obtained for cancer histology diagnosis and cancer biomarker analysis [8]. This study validated significantly high mRNA and protein levels of UBE2C in breast MC lesions, with no difference by age, BI-RAD category or pathology type or cancer stage. Knockdown of UBE2C expression by shRNA interference demonstrated the carcinogenesis role of UBE2C in proliferation, migration, and survival in breast cancer cell activities. As well, UBE2C expression was associated with that of selected oncogenic or metastasis genes. Our data suggest that UBE2C may be a candidate marker for diagnosis of nonpalpable breast lesions but not benign or malignant breast tumors. Suppression of UBE2C may be a potential therapy target in breast cancer.

UBE2C was highly expressed in MC but not non-MC lesions in mammography-guided core biopsies, which indicates the significant change in level of this oncogenic molecule in mammography-identified abnormal lesions. Therefore, the core biopsy is a valuable and important specimen in early cancer detection. UBE2C as a suggested malignant breast-cancer biomarker was evaluated in paraffin-embedded surgical specimens in previous studies [16], [21], [27], [28]. Here, we found UBE2C highly expressed in both malignant and benign breast cancer lesions, which suggests that UBE2C induction can be associated with abnormal cell growth. However, single-factor detection of UBE2C might be not sufficient to identify the tumor type or tumor progression in core biopsy specimens. Therefore, the diagnostic specificity is a concern when using UBE2C as a major biomarker to differentiate early malignant breast cancer from benign carcinoma. Previous studies suggested that UBE2C expression was accompanied by that of other biomarkers such as AZGP-1, prolactin-inducible protein, and S100A8 [29] or with pituitary tumor-transforming 1 (PTTG1), Survivin and thymidin kinase 1 [30], which might improve outcome prediction in breast cancer. Furthermore, UBE2C as a biomarker of efficacy of cancer chemotherapy should be explored.

We found that UBE2C expression was associated with the expression of the tumor initiator HER2, and MC lesions with high mRNA expression of HER-2 also showed high mRNA expression of UBE2C in breast core biopsies, which is consistent with previous observations in breast tumor tissue and cells [21], [28]. Importantly, the mRNA expression of the oncogene KRAS, a downstream factor of EGFR/HER2 [31], was altered in UBE2C–knocked-down MCF-7 cells. An as-yet unrevealed cross-regulation mechanism may exist among UBE2C, HER2 and KRAS. UBE2C expression was associated with that of the selected metastasis-related genes VEGF, CXCL-4, CCL5, NEDD9 and RHoC, in MCF-7 cells, so UBE2C may be involved in breast cancer metastasis. High expression of UBE2C was found in metastasized node-positive breast cancer [27].

Downregulating UBE2C expression with siRNA oligonucleotides inhibited cancer cell proliferation, arrested cells at S and G/M phases of the cell cycle and led to cell death [32], [33]. UBE2C siRNA-mediated cell apoptosis was enhanced by combined treatment with an agonist of the TNF-related apoptosis-inducing ligand (TRAIL) receptor [34]. In this study, we used retroviral vector-derived UBE2C shRNA to knock down UBE2C expression, which confirmed the important role of UBE2C in cell growth, migration and colony formation. We further demonstrated that UBE2C shRNA triggered cell death by activating an apoptosis pathway, which suggested the potential target of UBE2C for therapeutic intervention in cancer. In addition, our retrovirus-delivered shRNA for UBE2C suppression would be advantageous in anti-cancer study in a xenograft model *in vivo* [35], [36].

Development of a specific inhibitor of UBE2C would be a great benefit to cancer therapy. For instance, a UBE2C transcription inhibitor, CCI-779 [37], and a UBE2C-proteasome inhibitor, Bortezomib [38], blocked cell cycle progression in prostate cancer cells and colorectal carcinoma, respectively. In addition, cells with UBE2C knockdown sensitized breast cancer cells to radiation, doxorucibin and the hormone-blocking agents tamoxifen and letrozole [39]. Bioinformatic docking analysis identified certain small-molecule inhibitors against UBE2C including 2,4-diimino-1-methyl-1,3,5-triazepan-6-one, sulfuric acid compound with 5,6-diamino-2,4-pyrimidinediol (1∶1) and 7-alpha-d-ribofuranosyl-2-aminopurine-5′-phosphate [40]. For therapeutic application, these candidates require further study to confirm their inhibition activity of UBE2C.

In conclusion, we demonstrated that UBE2C biomarker expression in core biopsy specimens from mammography provides information to evaluate breast lesions or early breast carcinoma. Development of a UBE2C detection tool and targeting drug therapy would be of benefit to clinical practice in breast cancer diagnosis and treatment.

## Supporting Information

Figure S1 **Association of mRNA expression of UBE2C and other breast cancer markers.** The HER2, ER and PR expression of breast biopsies was determined by immunohistochemical staining in routine clinical practice. (**A**) HER2^low^ (histology score 0-1+), N = 3; HER2^high^ (score 2+-4+), N = 11. (**B**) ER1^low^ (score 1+), N = 4, ER^high^ (score 3+), N = 8. (**C**) PR^low^ (score 1-2+), N = 6, PR^high^ (score 3+-4+), N = 6. Data are mean±SEM.
(TIF)

Table S1 **qPCR primers used in this study.**
(DOC)
