# Supplementary figures and images for: Ubiquitin-Conjugating Enzyme UBE2C Is Highly Expressed in Breast Microcalcification Lesions
Source: PLoS One. 2014 Apr 3;9(4):e93934. doi: 10.1371/journal.pone.0093934 (PMC3974821; doi:10.1371/journal.pone.0093934)

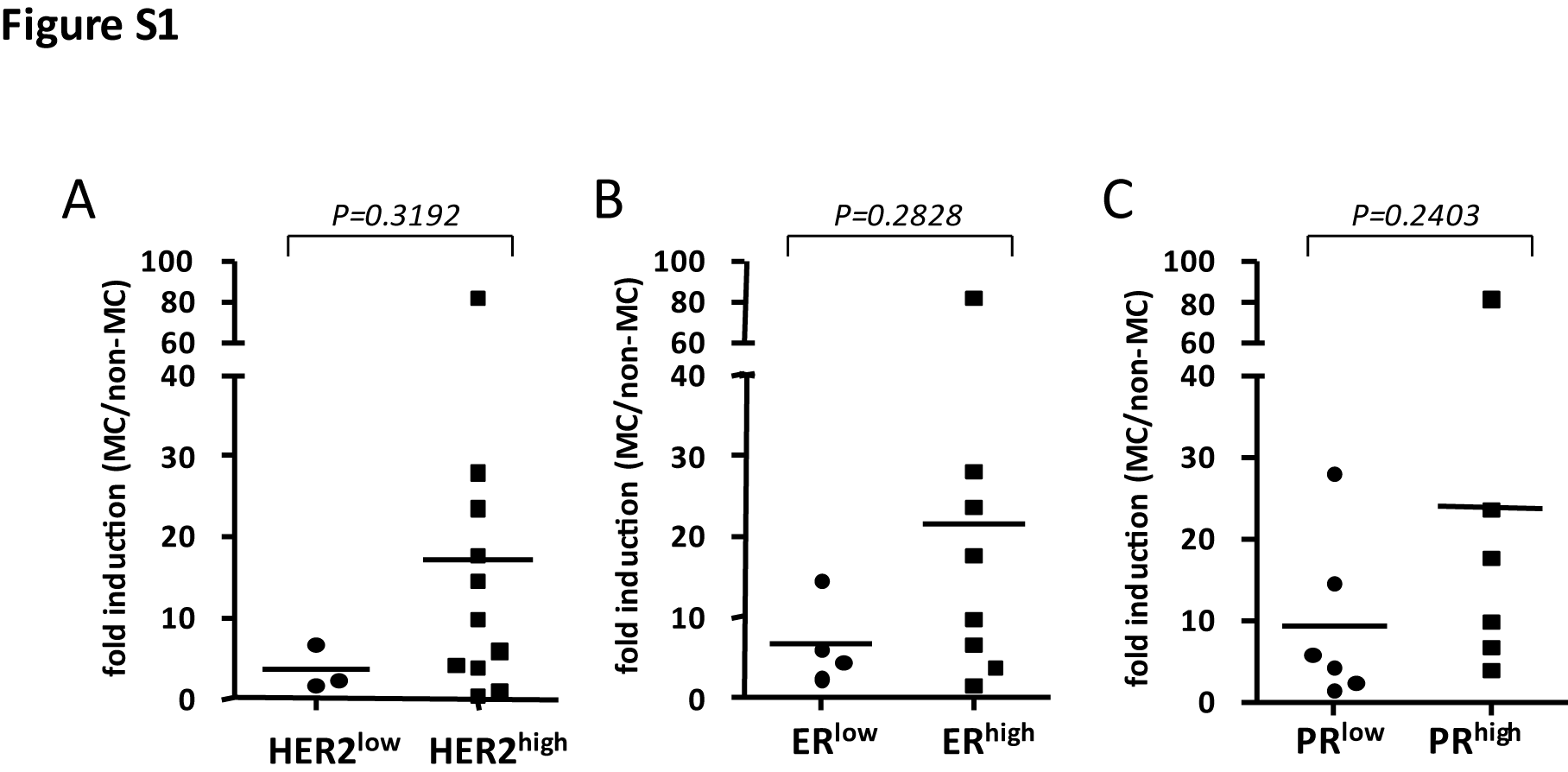

Supplement: Figure S1 — Association of mRNA expression of UBE2C and other breast cancer markers. The HER2, ER and PR expression of breast biopsies was determined by immunohistochemical staining in routine clinical practice. (A) HER2low (histology score 0-1+), N = 3; HER2high (score 2+-4+), N = 11. (B) ER1low (score 1+), N = 4, ERhigh (score 3+), N = 8. (C) PRlow (score 1-2+), N = 6, PRhigh (score 3+-4+), N = 6. Data are mean±SEM. (TIF) [file pone.0093934.s001.tif]
