# Supplementary material for: Ubiquitin-Conjugating Enzyme UBE2C Is Highly Expressed in Breast Microcalcification Lesions
Source: PLoS One. 2014 Apr 3;9(4):e93934. doi: 10.1371/journal.pone.0093934 (PMC3974821; doi:10.1371/journal.pone.0093934)
Supplement: Table S1 — qPCR primers used in this study. (DOC) [file pone.0093934.s002.doc]

## Table S1. qPCR primers used in this study.

| **Gene** | **Accession number of reference gene** | **Primer sequences** |
| --- | --- | --- |
| UBE2C | NM_007019.2 | F’- TGCCCTGTATGATGTCAGGA |
|  |  | R’- GGGACTATCAATGTTGGGTTCT |
| HER2 | NM_000448.2 | F’- CCCAACCAGGCGCAGAT |
|  |  | R’- GTGCCAAAAGCGCCAGAT |
| KRAS | NM_001130089.1 | F’- GGCAGCGAGCCGAAACT |
|  |  | R’- GGCAGAGCACCCTGGAACTA |
| VEGF | NM_001025366.2 | F’- CCCACTGAGGAGTCCAACATC |
|  |  | R’- GCTGGCCTTGGTGAGGTTT |
| CXCR4 | NM_001008540.1 | F’- AAATCTTCCTGCCCACCATCT |
|  |  | R’- ACCAATCCATTGCCCACAAT |
| CCL5 | NM_002985.2 | F’- CATCTGCCTCCCCATATTCCT |
|  |  | R’- GCGGGCAATGTAGGCAAA |
| NEDD9 | NM_006403.3 | F’- CGGCAGTTGCTGTGCTTCTA |
|  |  | R’- AATGGCGTTGAGAAGGGAAA |
| RHoC | NM_175744.4 | F’- AAGTGGACCCCAGAGGTGAAG |
|  |  | R’- TTATTCCCCACCAGGATGATG |
| HPRT | NM_000194.2 | F’- GAACGTCTTGCTCGAGATGTGA |
|  |  | R’- GAGCACACAGAGGGCTACAATG |
